# The effects of three different low-volume aerobic training protocols on cardiometabolic parameters of type 2 diabetes patients: A randomized clinical trial

**DOI:** 10.3389/fendo.2023.985404

**Published:** 2023-01-23

**Authors:** Paulo Gentil, Lucas Raphael Bento e Silva, Daniela Espíndola Antunes, Luciana Barbosa Carneiro, Claudio Andre Barbosa de Lira, Gislene Batista, Jordana Campos Martins de Oliveira, John Sebastião Cardoso, Daniel CostaCosta Souza, Ana Cristina Silva Rebelo

**Affiliations:** ^1^ College of Physical Education and Dance, Federal University of Goiás, Goiânia, Brazil; ^2^ Hypertension League, Federal University of Goiás, Goiânia, Brazil; ^3^ Instituto VIDA, Brasilia, Brazil; ^4^ Faculty of Medicine, Federal University of Goiás, Goiânia, Brazil; ^5^ Hospital das Clínicas, Federal University of Goiás, Goiânia, Brazil; ^6^ Banco de Olhos Foundation, Goiânia, Brazil; ^7^ Faculty of Nutrition, Federal University of Goiás, Goiânia, Brazil

**Keywords:** diabetes mellitus, high-intensity interval training (HIIT), aerobic exercise (AE), glucose control, exercise is medicine

## Abstract

**Objective:**

To compare the effects of different aerobic training protocols on cardiometabolic variables in patients with type 2 diabetes mellitus (T2DM).

**Methods:**

This study was a parallel clinical trial. Fifty-two men and women with T2DM (>40 years) were randomly allocated into three groups, and 44 (22 males/22 females) were included in the final analysis. Exercise intensity was based on the speed corresponding to the maximum oxygen consumption (v $\dot{V}$ O_2_max). Moderate intensity continuous training (MICT) involved 14 minutes at 70% of v $\dot{V}$ O_2_max; short interval high-intensity interval training (S-HIIT) consisted of 20 bouts of 30 seconds at 100% of *V*˙O_2_max with 30 seconds passive recovery; long interval high-intensity training (L-HIIT) consisted of 5 bouts of 2 minutes at 100% of v $\dot{V}$ O_2_max with 2 minutes passive recovery. Training protocols were performed on a motorized treadmill two times per week for eight weeks. Glycated hemoglobin (Hb1Ac), total cholesterol, triglycerides, resting systolic blood pressure (SBP), resting diastolic blood pressure (DBP), resting heart rate (resting HR) and maximum oxygen consumption (*V*˙O_2_max) were measured before and after the exercise intervention. The study was registered on the Brazilian clinical trial records (ID: RBR45 4RJGC3).

**Results:**

There was a significant difference between groups for changes on $\dot{V}$ O_2_max. Greater increases on $\dot{V}$ O_2_max were achieved for L-HIIT (p = 0.04) and S-HIIT (p = 0.01) in comparison to MICT group, with no significant difference between L-HIIT and S-HIIT (p = 0.9). Regarding comparison within groups, there were significant reductions on HbA1c and triglycerides levels only for L-HIIT (p< 0.05). $\dot{V}$ O_2_max significantly increased for both L-HIIT (MD = 3.2 ± 1.7 ml/kg/min, p< 0.001) and S-HIIT (MD = 3.4 ± 1.7, p< 0.001). There was a significant reduction on resting SBP for L-HIIT group (MD = -12.07 ± 15.3 mmHg, p< 0.01), but not for S-HIIT and MICT. There were no significant changes from pre- to post-training on fasting glycemia, total cholesterol, HDL, LDL, resting HR and resting DBP for any group (p > 0.05).

**Conclusion:**

Low-volume HIIT promoted greater improvements in cardiorespiratory capacity in comparison with low-volume MICT, independent of the protocols used. There were no other differences between groups. All protocols improved at least one of the variables analyzed; however, the most evident benefits were after the high-intensity protocols, especially L-HIIT.

## 1 Introduction

Type 2 diabetes mellitus (T2DM) is one of the leading causes of disability worldwide (1). T2DM is associated with increased mortality and reduced quality of life and might have serious complications, such as retinopathy, cardiovascular diseases and limb amputations (2). Therapeutic recommendations for T2DM aim to control glycemia, lipidemia, blood pressure (BP), body mass and promote lifestyle changes, such as increase physical activity levels (3). In this sense, aerobic training is considered an effective strategy for T2DM prevention and treatment (4, 5). Traditionally, performing at least 150 min/week of moderate (MICT) to vigorous intensity continuous training distributed at a minimum of three non-consecutive day during the week has been recommended to manage T2DM (6). However, there is evidence that high-intensity interval training (HIIT) might provide superior benefits on a variety of cardiometabolic risk factors in comparison to MICT (7, 8), leading physical activity guidelines to suggested the use of HIIT for managing T2DM (9).

HIIT is a type of aerobic training that consists of performing high intensity exercise bouts alternated with passive or active recovery periods (10). There are many different types HIIT, which might result in different physiological and perceptual responses (10–12). Among them, we can highlight the protocols involving shorter intervals (S-HIIT) and longer intervals (L-HIIT) (13).

Kilpatrick et al., (14) compared three work-matched HIIT protocols performed at the same intensity, but with different interval durations (120 s vs. 60 s vs. 30 s) in healthy young people. According to the results, longer intervals were associated with greater cardiovascular stress and higher discomfort (14). Similarly, Naves et al., (11) and Silva et al., (15) found that L-HIIT promoted greater cardiovascular stress in comparison with S-HIIT in healthy young men, and women with metabolic syndrome, respectively. Interestingly, most studies involving HIIT in T2DM used long intervals (≥2 minutes), which could be potentially dangerous due to the greater cardiovascular stress, especially if we consider that most T2DM patients have increased cardiovascular risks (16). However, while L-HIIT protocols may lead to increased cardiovascular stress, it also results in higher work performed, higher oxygen consumption and higher heart rate (HR), which can make them more efficient for promoting cardiometabolic adaptations (7, 17, 18).

In this context, it is important to determine whether the use of different HIIT protocols could affect cardiometabolic adaptations in T2DM in order to allow an adequate cost benefit analysis. Therefore, the aim of the present study was to investigate the effects of three different types of aerobic training protocols on cardiometabolic parameters in people with T2DM.

## 2 Material and methods

### 2.1 Study design

This study is a parallel randomized clinical trial that involved individuals with T2DM of both sex that performed different aerobic training protocols, two times per week for eight weeks. The study was performed at the Hospital of the Federal University of Goias (Goiania, Brazil), approved by the relevant Human Research Ethics Committee (Protocol No. 2,667,732, CAAE No. 54522016.6.0000.5083) and registered on the Brazilian clinical trial records (ID: RBR-4RJGC3). All participants gave written informed consent in accordance with the Declaration of Helsinki. Participants were assigned by randomization to one of three groups using a specialized website (www.random.org): L-HIIT, S-HIIT, or MICT.

Before and after the training period, participants were evaluated for anthropometric measures, resting BP, cardiopulmonary exercise test and blood markers. Anthropometric measures included weight and height for calculating body mass index (BMI), as well as waist and hip circumference. Weight was measured using a digital scale (BC 553, Tanita^®^, USA), and height was measured using a portable stadiometer (Personal Caprice Portatil, Sanny^®^, Brazil). BMI was calculated using the following formula [BMI = weight (kg)/height (m)²]. BP and HR were measured at sitting position after 10 minutes of rest according with Seventh Brazilian Arterial Hypertension Guideline using oscilometric method (Omron HEM-705) (19). Complementary results of the current experiment have been published previously (20).

All experimental procedures were carried out in the morning, with relative humidity between 40 and 60%, and temperature between 22 and 24°C, according to American College of Sports Medicine guidelines (ACSM) (21). Participants received orientation to not drink or eat products containing alcohol or caffeine 24 hours before and on the day of the tests, to not perform physical exercises or strenuous activities on the day before exercise, to have a light meal at least 2 hours before the tests, to wear clothes suitable for physical activity practice and to keep their usual diets habits during the intervention period.

### 2.2 Participants

Sixty participants with T2DM were recruited from 3rd Diabetes Task Force promoted by the Eye Bank Foundation of Goiás. To be included in the study, participants had to be 40 years old or more, diagnosed with T2DM, have a fasting glycemia above 126 mg/dL and/or glycated hemoglobin greater than 6.4%, and to not participate in other physical training program. Patients were excluded if they were active smokers or had myocardial revascularization, arrhythmias and frequent extrasystoles at rest or during physical exertion, unstable angina, obstructive pulmonary disease, neoplasm, renal or liver failure, orthopedic limitations, and uncontrolled hypothyroidism and cardiovascular diseases at moderate and high risk (classes C and D), according to the criteria American Heart Association (22). To be included in the final analyses, participants had to perform more than 85% of all training sessions.

**Figure 1:**
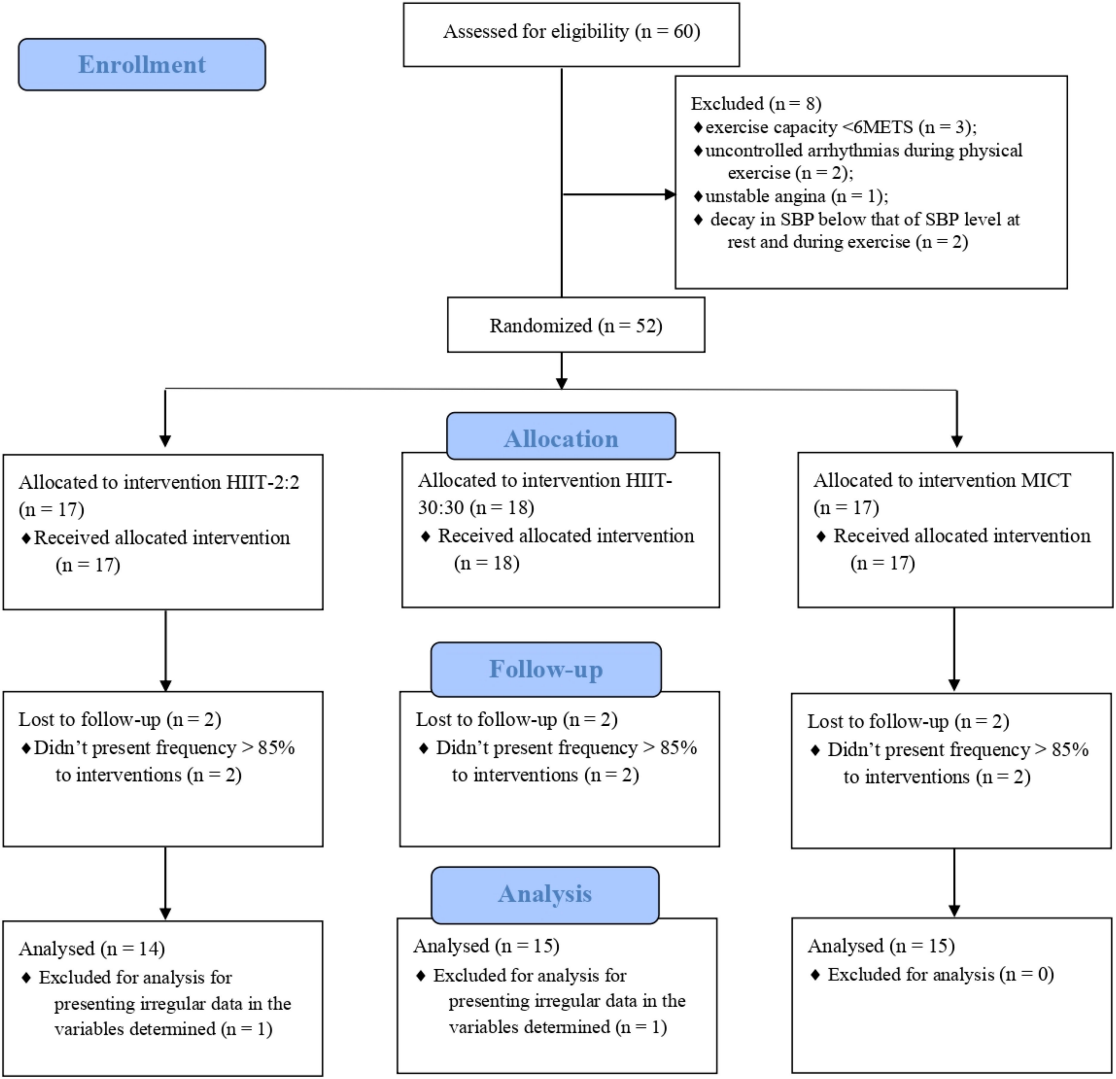
Study flow according to CONSORT recommendations. MICT, moderate intensity continuous training; HIIT, high-intensity interval training; SBP, systolic blood pressure.

After cardiopulmonary exercise tests, eight patients were excluded, including three with an exercise capacity<6 METS, two with uncontrolled arrhythmias during physical exertion, one with unstable angina, and two with a reduction in SBP during exercise to lower levels than resting SBP. Two patients were excluded from the final analyses because they presented irregular data for determined variables. The enrolment process until participant’s inclusion in final analyses is describe in **Figure 1**.

The was no *a priori* definition of sample size. However, the total number of 60 participants was considered adequate because it provided a power > 0.9 to detect an effect size of 0.5 (G*Power 3.1, Germany).

### 2.3 Medication record

Medication use was self-reported according to **Table 1** and there was no change in the medications of the patients during the intervention period.

**Table 1:** Class of medicines used by patients.

Medication	Long HIIT (n =15)	Short HIIT (n =15)	MICT (n =14)
Biguanides	11	12	10
Sulfonylureas	1	–	3
SGLT2 inhibitors	2	–	1
DPP-4 inhibitors	1	1	1
GLP-1 analogue	–	1	2
Pioglitazones	–	1	1
Insulin	8	7	5
Anti-Hypertensive	15	15	14
Anticholesterolemic	13	12	11

HIIT, high-intensity interval training; MICT, moderate intensity continuous training; SGLT2, Sodium glucose cotransporter 2; DPP-4, Dipeptidyl peptidase-4; GLP-1, Glucagon-like peptide-1.

### 2.4 Biochemical analyses

At the first visit to the laboratory, 4 mL of whole blood was collected by the vacuum method into EDTA tubes (Plastilab, Brazil) from the antecubital vein after approximately 12 h overnight fasting. Homogenized whole blood was used for preparation and processing of samples.

The following analyzes were performed for glucose metabolism: fasting glucose by enzymatic method using commercial kits (Labtest, Brazil), fasting insulin by chemiluminescence and HbA1c by immunoassay. This method is certified by the National Glycohemoglobin Standardization Program (23).

Lipid profile analysis involved total cholesterol by enzymatic system (reaction of the endpoint), HDL by System for direct homogeneous determination of HDL cholesterol in serum, and triglycerides by enzymatic system, by end point reaction, all using commercial kits (Labtest, Brazil).

### 2.5 Cardiopulmonary exercise test (CPET)

CPET involved an incremental ramp type protocol performed on a treadmill (Micromed^®^, Centurion 200, Brasília, Brazil) coupled to a computer. The test started with a two-minute warm-up with no inclination and speed correspondent to 50% of the initial values predicted for age and sex. Every 15 seconds of warm-up, speed increased by 0.5 km/h.

After warm-up, treadmill speed was set according to age and gender, following the recommendations of the Brazilian Society of Cardiology. The speed was then increased by 0.1 km/h every 10, 20 or 30 seconds until voluntary exhaustion, and participants were verbally encouraged to give their maximum effort. The test lasted between 8 to 12 minutes and ended when participants met the criteria for test interruption according to ACSM guideline (21).

Volunteers were instructed to wear comfortable clothes and avoid vigorous physical exercise (24 hours before the test), alcohol consumption (12 hours before the test) and caffeine (3 hours before the test). CPET was performed in the morning, to avoid the influence of the circadian rhythm. Ambient temperature (22°C - 24°C), relative humidity (40% - 60%) and lighting were controlled according to the preliminary conditions (23). Gas analysis was performed using a Cortex^®^ analyzer (Metalyser II, Rome, Italy). Equipment calibration was performed for barometric pressure, ambient gas, gas mixture, flow and volume, as per the manufacturer’s recommendations.

HR, BP, rating of perceived exertion (RPE) and ventilatory parameters (peak, carbon dioxide production, respiratory oxygen equivalent, respiratory equivalent of the ventilatory threshold) were monitored and registered during the test. RPE was evaluated according to the Borg 0-10 (27). The instrument presents numerical classification from “0” to “10”, indicating low and maximum intensity effort, respectively. The values indicated by the volunteers were recorded at the end of each minute of the test (5th to 16th minute). Heart rate (HR) was measured by a cardiac monitor (Polar v800, Finland) during the test and up to 10 minutes of rest after its end, with the volunteer in the sitting position. Cardiac monitor was fixed in the chest and with simultaneous transmission to a watch. Data were later transferred and recorded in a specific software (Polar Flow, Finland) for proper analysis.

### 2.6 Exercise protocols

The protocols were customized with individualized monitored of HR, BP and v $\dot{V}$ O_2_max achieved in the (CPET). Their respective exercise intensities were adapted from previous studies (11, 15), and were matched by the product (time * % v $\dot{V}$ O_2_max).

Exercise session started with a 2 min warm-up and ended with a 2 min cooldown at 50% of v $\dot{V}$ O_2_max. MICT consisted of continuous walking/running at 70% of v $\dot{V}$ O_2_max for 14 minutes. L-HIIT consisted of 5 bouts of 2 minutes walking/running at 100% of v $\dot{V}$ O_2_max interspersed by a passive recovery of 2 minutes. S-HIIT consisted of 20 bouts of 30 seconds walking/running at 100% of v $\dot{V}$ O_2_max interspersed by a passive recovery for 30 seconds. All protocols were performed on a motorized treadmill (Micromed^®^, *Centurion 200*, Brasília, Brazil).

Exercise intensity was adjusted using perceived of exertion as previously suggested (24, 25).

### 2.7 Statistical analyses

Data normality and homogeneity were tested using the Shapiro-Wilk and Levene test, respectively. Post-training values were compared between groups using analysis of covariance (ANCOVA) with pre-training values as covariates. When a significant effect was identified, *Bonferroni’s post hoc* was used to identify were differences occurred. Paired T-test was used to compare within groups differences for HbA1c, fasting glycemia, total cholesterol, triglycerides, HDL, LDL, resting SBP, resting DBP, resting HR and $\dot{V}$ _O2_max using pre- and post-training values.

All analyses were performed using SPSS statistical package (Statistical Package for Social Sciences Chicago, IL, USA) version 20.0 for Windows. Results are expressed as mean and standard deviation, and the accepted level of significance was (p< 0.05).

## 3 Results

The characteristics of participants are described in **Table 2**.

**Table 2:** Characteristics of participants.

	MICT (n = 9M;5F)	L-HIIT (n = 6M;9F)	S-HIIT (n = 7M;8F)	p-value
Age (years)	54.6 ± 8.9	57.3 ± 8.9	55.7 ± 7.4	0.69
Weight (kg)	80.7 ± 14.5	76.3 ± 16.9	79.5 ± 11.0	0.69
Height (cm)	165.4 ± 11.0	163.1 ± 8.3	165.5 ± 6.7	0.68
BMI	29.4 ± 4.9	28.5 ± 4.9	28.9 ± 3.6	0.85

BMI, body mass index; training; S-HIIT, short high-intensity interval training; L-HIIT, long high-intensity interval training; MICT, moderate intensity continuous; M, Male; F, Female. Data presented as mean ± standard deviation.

There were significant difference between groups for changes on $\dot{V}$ O_2_max, with greater increases on $\dot{V}$ O_2_max for L-HIIT (p = 0.04) and S-HIIT (p = 0.01) in comparison with MICT group. There was no significant difference between L-HIIT and S-HIIT (p = 0.9). Pre- and post-training values and comparisons between and within groups are presented in **Table 3**.

**Table 3:** Comparison between and within groups for dependent variables.

Variables	Pre	Post	Mean difference	p-value within groups	p-value between groups
	mean ± SD	mean ± SD			
HbA1c (%)					
MICT (n=14)	8.5 ± 2.4	7.9 ± 1.2	-0.59 ± 1.7	0.21	0.28
L-HIIT (n=15)	9.9 ± 2.8	8.1 ± 2.1	-1.80 ± 1.8	**<0.01**	
S-HIIT (n=15)	9.6 ± 1.9	8.7 ± 1.6	-0.92 ± 2.0	0.09	
Fasting glycemia (mg/dL)					
MICT	129.8 ± 57.1	126.7 ± 54.6	-3.07 ± 28.0	0.69	0.10
L-HIIT	145.5 ± 66.0	140.1 ± 69.0	-5.33 ± 36.3	0.58	
S-HIIT	142.7 ± 63.0	136.9 ± 57.9	-5.83 ± 29.6	0.46	
Triglycerides (mg/dL)					
MICT	159.6 ± 33.2	164.9 ± 40.3	5.36 ± 31.9	0.54	0.06
L-HIIT	164.9 ± 84.1	141.1 ± 48.1	-23.80 ± 39.6	**0.04**	
S-HIIT	185.5 ± 75.9	162.3 ± 58.3	-23.19 ± 49.0	0.09	
Total cholesterol (mg/dL)					
MICT	172.9 ± 44.7	75.1 ± 36.2	2.21 ± 43.7	0.85	0.94
L-HIIT	199.5 ± 65.2	190.9 ± 53.6	-8.60 ± 46.7	0.49	
S-HIIT	188.3 ± 38.6	180.2 ± 42.5	-8.05 ± 32.1	0.35	
HDL (mg/dL)					
MICT	44.4 ± 8.9	43.5 ± 9.2	-4.45 ± 1.2	0.49	0.16
L-HIIT	43.1 ± 10.1	47.1 ± 13.3	3.93 ± 8.9	0.11	
S-HIIT	46.4 ± 8.0	45.9 ± 7.5	-0.62 ± 6.8	0.72	
LDL (mg/dL)					
MICT	98.3 ± 40.7	98.6 ± 32.1	0.29 ± 38.9	0.98	0.27
L-HIIT	107.7 ± 43.4	113.9 ± 34.2	6.21 ± 23.2	0.32	
S-HIIT	116.7 ± 38.1	104.3 ± 35.1	-12.39 ± 27.0	0.10	
Resting SBP (mmHg)					
MICT	126.1 ± 19.8	130.5 ± 13.4	4.43 ± 13.1	0.23	0.27
L-HIIT	145.5 ± 25.5	133.5 ± 17.6	-12.07 ± 15.3	**<0.01**	
S-HIIT	138.6 ± 15.8	131.3 ± 17.6	-7.33 ± 17.1	0.12	
Resting PAD (mmHg)					
MICT	82.6 ± 7.0	86.3 ± 6.2	3.64 ± 7.6	0.10	0.21
L-HIIT	86.6 ± 10.1	84.3 ± 6.0	-2.33 ± 9.7	0.37	
S-HIIT	84.3 ± 9.7	87.7 ± 10.2	3.33 ± 8.6	0.16	
Resting HR (bpm)					
MICT	75.9 ± 11.3	68.2 ± 9.5	-7.71 ± 10.9	0.02	0.07
L-HIIT	77.5 ± 10.3	75.1 ± 8.5	-2.40 ± 6.7	0.19	
S-HIIT	71.7 ± 12.0	72.3 ± 15.0	0.6 ± 0.1	0.80	
$\dot{V}$ O_2_max (ml/kg/min)					
MICT	22.6 ± 8.9	23.4 ± 10.7	0.86 ± 3.8	0.41	0.01*^,#^
L-HIIT	22.4 ± 5.6	25.6 ± 6.3	3.2 ± 1.7	**<0.001**	
S-HIIT	19.7 ± 3.1	23.1 ± 3.7	3.4 ± 1.7	**<0.001**	

MICT, moderate intensity continuous training; L-HIIT, long high intensity interval training; S-HIIT, short high intensity interval training; HbA1c, glycated hemoglobin; HDL, high-density lipoprotein; LDL, low-density lipoprotein; SBP, systolic blood pressure; DBP, diastolic blood pressure; HR, heart rate; $\dot{V}$ O_2_max, maximum oxygen consumption.

* L-HIIT > MICT.

# S-HIIT > MICT. Bold values mean p<0.05.

In comparison with pre-training values, there was a significant reduction on HbA1c levels for L-HIIT (mean difference [MD] = -1.8 ± 1.8%, p< 0.01), while no significant change was achieved for S-HIIT (MD = -0.92 ± 2.0%, p = 0.09) and MICT (MD = -0.59 ± 1.7%, p = 0.2). Triglycerides levels significantly reduced after L-HIIT (MD = -23.80 ± 39.6 mg/dL, p = 0.04), with no changes for S-HIIT (MD = -23.19 ± 49.0 mg/dL, p = 0.09) and MICT (MD = 5.36 ± 31.9 mg/dL, p = 0.5). There were no significant changes from pre- to post-training on fasting glycemia, total cholesterol, HDL, and LDL (p > 0.05).

There was a significant reduction on resting SBP for L-HIIT group (MD = -12.07 ± 15.3 mmHg, p< 0.01), but not for S-HIIT (MD = -7.33 ± 17.1 mmHg, p = 0.1) and MICT (MD = 4.43 ± 13.1 mmHg, p = 0.2). $\dot{V}$ O_2_max significantly increased for both L-HIIT (MD = 3.2 ± 1.7 ml/kg/min, p< 0.001) and S-HIIT (MD = 3.4 ± 1.7, p< 0.001), but not for MICT (MD = 0.86 ± 3.8, p = 0.4). There were no significant changes from pre- to post-training for resting HR and resting DBP for any group (p > 0.05).

## 4 Discussion

The present study investigated the effects of three aerobic training protocols (L- HIIT, S-HIIT, and MICT) on cardiometabolic parameters of patients with T2DM. The recommendation of a minimum of 150 min per week of moderate intensity physical activities for blood glucose control and cardiovascular health is still predominant (26). However, our study demonstrated that the performance of 20 min high intensity exercise per week was sufficient to improve cardiorespiratory and metabolic fitness in patients with T2DM. These findings are particularly important since time-efficient exercise strategies emerge as promising alternatives to improve exercise adherence. Moreover, this is one of the first studies to compare HIIT protocols involving different interval lengths on metabolic parameters in T2DM patients. The present study found a significant reduction in HbA1c with large effect after L-HIIT, corroborating the idea that exercise intensity can play an important role in managing T2DM (27). Moreover, the use of HIIT protocols involving longer intervals might be preferred for blood glucose control in this population. These improvements in Hb1Ac can be explained by the increase in GLUT4 protein due to a higher concentration of calcium led by increased exercise intensity, resulting in protein translocation to the cell membrane and increasing glucose uptake by muscle cells (28–30). L-HIIT might also have led to a higher glycogen depletion, which is associated with improved glucose uptake (31, 32). A previous study demonstrated that PGC-1α increase (important protein for metabolic gene activation necessary for the use of substrate and mitochondrial biogenesis) occurs after HIIT, but not after MICT (33).

Previous studies have shown 15 to 20% reductions in cardiovascular events when HbA1c is reduced by 1% (34, 35). In addition, this reduction on HbA1c for L-HIIT group observed after 8 weeks of training is apparently higher than the observed after long-term (> 12 weeks) hypoglycemic drug treatment and insulin use (ranging from 0.6-0.8%) (45). In agreement with the current study, Winding et al., (36) found greater reductions on Hb1Ac after 11 weeks of HIIT (i.e., 10 bouts of 1 minute at 95% of peak workload interspersed by 1 minute of active rest) when compared to endurance training in individuals with T2DM. Moreover, HIIT has been shown to promote a rapid increase in skeletal muscle oxidative capacity, insulin sensitivity and glycemic control in adults with T2DM (7, 37, 38). When compared to MICT, HIIT programs have promoted greater improvements in HbA1c, fasting glycemia and other risk factors for T2DM (34, 35).

Although no significant difference was found for lipid profile between groups, there was a trend to decrease triglycerides and a large effect was found between pre and post intervention for both HIIT protocols [L-HIIT [-0.57 (wide)]; S-HIIT [-0.53 (wide)]. It is important to acknowledge that improvements in triglyceride and HDL levels are important due to the associations between dyslipidemia and cardiovascular diseases (39). The effects of HIIT on lipid profile in patients with T2DM have been inconsistent in the literature. There are studies showing improvements only in LDL cholesterol (40), only in HDL (41), or absence of alteration (42). The same studies also showed no effect of HIIT on triglycerides in patients with T2DM (40–42).

L-HIIT showed a greater reduction in resting SBP when compared to MICT (p = 0.018; d = 1.56 [large effect]). Although several studies have shown BP reductions through exercises (43), the reduction only in L-HIIT can be explained by the difference in baseline values (MICT 125 [25]mm/Hg; L-HIIT 147 [45]mm/Hg; S-HIIT 135 [20]mm/Hg), since greater reductions are seen in people with higher BP levels (44). Although there seems to be an agreement on the effectiveness of exercise to reduce BP (45), the effectiveness of aerobic exercise to reduce systolic and diastolic arterial pressure in patients with T2DM is still debated, with previous studies showing contradictory results (6, 46). Previous studies showed reductions in SBP after HIIT (~ 13 mmHg) (41) compatible with our results, revealing a potential for HIIT for controlling cardiovascular risks (47). $\dot{V}$ O_2_max increases after HIIT is in accordance with the results of previous studies (48–50). Between group comparisons showed that the increases in $\dot{V}$ O_2_max were larger in the L-HIIT and S-HIIT than MICT. This is in accordance with the suggestion that HIIT are more beneficial for the cardiorespiratory system. The absence of an increase $\dot{V}$ O_2_max in the MICT was unexpected but could be associated with the changes in HbA1c since there are studies showing an association between $\dot{V}$ O_2_max increase and in HbA1c reduction (R = −0.52, p<0.01). According to these findings, approximately 25% of the reductions in HbA1c may be related to the increase in $\dot{V}$ O_2_max (51). $\dot{V}$ O_2_max is an objective and independent indicator of cardiovascular risk and considered the most important physical conditioning variable (52, 53). Cardiorespiratory fitness is inversely associated with all-cause mortality and an increase of 1-2 METs (MET = 3.5ml O2/Kg/min) reduces 10% to 30% the risk of cardiovascular events (54). Although evidence for an optimal exercise intensity is still uncertain, it has been suggested that only exercise with intensity close to $\dot{V}$ O_2_max allows the recruitment of large motor units (i.e., type II muscle fibers) (55, 56) and induces high cardiac output, which might be important for long term VO2max improvements (57).

One important limitation of the present study is the absence of dietary control. However, participants were oriented to not change their diet. We believe that these limitations do not prevent the conclusions of the study from being elaborated. In addition, due the lack of control group, it was not possible to know whether changes observed for exercising groups were different when compared to non-exercise conditions.

## 5 Conclusion

Low-volume HIIT promoted greater improvements in cardiorespiratory capacity in comparison with low-volume MICT, independent of the protocols used. There were no other difference between groups. All protocols improved at least one of the variables analyzed; however, the most evident benefits were after the high-intensity protocols, especially L-HIIT. Therefore, it will be up to the professional to analyze their patients individually to propose the best intervention for each case, within an appropriate cost-benefit perspective.

## Data availability statement

The raw data supporting the conclusions of this article will be made available by the authors, under reasonable request.

## Ethics statement

The studies involving human participants were reviewed and approved by Federal University of Goias Ethics Committee. The patients/participants provided their written informed consent to participate in this study.

## Author contributions

PG conceived and designed the research. LS, JC, and JSC performed experiments. DS and PG analyzed data, interpreted results of experiments, and drafted manuscript. DS and AR edited and revised manuscript. All authors contributed to the article and approved the submitted version.
